# Fact or fiction: updates on how protein-coding genes might emerge *de novo* from previously non-coding DNA

**DOI:** 10.12688/f1000research.10079.1

**Published:** 2017-01-19

**Authors:** Jonathan F Schmitz, Erich Bornberg-Bauer

**Affiliations:** 1 Institute for Evolution and Biodiversity, University of Muenster, Muenster, Germany

**Keywords:** De novo protein evolution, novel genes, de novo genes, gene emergence, protein-coding genes

## Abstract

Over the last few years, there has been an increasing amount of evidence for the *de novo* emergence of protein-coding genes, i.e. out of non-coding DNA. Here, we review the current literature and summarize the state of the field. We focus specifically on open questions and challenges in the study of *de novo* protein-coding genes such as the identification and verification of *de novo*-emerged genes. The greatest obstacle to date is the lack of high-quality genomic data with very short divergence times which could help precisely pin down the location of origin of a *de novo* gene. We conclude that, while there is plenty of evidence from a genetics perspective, there is a lack of functional studies of bona fide *de novo* genes and almost no knowledge about protein structures and how they come about during the emergence of *de novo* protein-coding genes. We suggest that future studies should concentrate on the functional and structural characterization of *de novo* protein-coding genes as well as the detailed study of the emergence of functional *de novo* protein-coding genes.

## Introduction

The question of how new genes come about has been a major research theme in evolutionary biology since the discovery that different species’ genomes contain varying numbers of genes. This question is difficult to answer, since emerging genes cannot easily be “caught in the act”. Ohno ^1^ gave the first comprehensive answer: new genes can emerge via the duplication of old genes. Consequently, gene duplication was thought to be the only mechanism of gene birth for many years ^2^. However, the discovery of so-called orphan genes in newly sequenced genomes raised doubt about the general validity of Ohno’s model of gene duplication. Orphan genes are genes that lack detectable homologs outside of a species or lineage. To explain the presence of orphans under the assumption that new genes emerge only via duplication, one has to assume gene loss in all other lineages or a phase of highly accelerated evolution that leads to the loss of detectable sequence similarity ^3^. Yet convergent gene loss in many independent lineages is unlikely — especially given the high number of orphan genes — and it is difficult to explain why so many genes would experience prolonged phases of accelerated evolution ^4^. On the contrary, it would be expected that genes that do not experience any selective pressure — which is required here for accelerated evolution — would be pseudogenized eventually, i.e. not be transcribed anymore.

These inconsistencies and further observations suggested that there could be other mechanisms of gene emergence ^5, 6^, for example *de novo* gene emergence, a process in which a new gene evolves from a previously non-genic sequence. The product of this process can be an RNA gene or a protein-coding gene. The possibility of *de novo* gene emergence has long been disputed, with many claiming that it is impossible for an intergenic, random open reading frame (ORF) to encode a functional protein (reviewed in 4, 7). But, despite these open questions regarding the exact mechanism of *de novo* gene birth, many recent studies report *de novo* emergence of protein-coding genes ^5, 6, 8– 19^.

**Figure 1.**
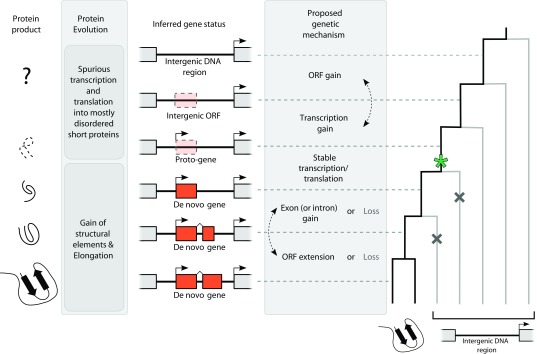
Schematic depiction of *de novo* protein-coding gene emergence. Shown is the hypothesis of a step-wise genic and structural maturation of an intergenic sequence towards a protein-coding gene. The steps are each shown as pictograms of protein and gene structure. An exemplary phylogenetic tree is shown to the right. The status of the protein/gene is projected onto the tree using grey, dotted lines. Gene emergence is depicted using a green star, gene loss using a grey X symbol. ORF, open reading frame.

In general, genes without detectable homologs can be summarized under the term *novel* genes. These genes can also be called *orphan* genes, or — more precisely — *species-/lineage-specific* genes. The term *de novo* describes a specific subclass of novel genes, namely genes emerging from non-genic sequences ^20^. Additionally, one has to discriminate between functional genes and other classes of sequences. A *de novo* transcript can be any species-specific transcript that is homologous to an intergenic sequence in outgroups. *De novo* transcripts can be seen as putative *de novo* genes (see also Figure 1). The term *protogene* also describes intergenic transcripts or ORFs that are situated on a continuum between non-genic sequences and functional genes ^21^ (see also Figure 1). At the genic end of the spectrum, the term *de novo* gene describes a functional gene that has emerged *de novo*. *De novo* genes can either code for a protein or be functional as RNAs ^22^. Here, we will use the term *de novo* gene to describe *de novo* genes of unknown coding status and *de novo* protein-coding gene to describe *de novo*-emerged genes that likely produce a functional protein product.

### Identification of *de novo* genes

The first step necessary to determine *de novo* status of a gene is to verify that no homologous sequences are present in outgroups. This homology search is often performed using BLAST or similar alignment search tools, for example against non-redundant protein databases containing all known protein sequences. Usually, an e-value cutoff between 10 ^−3^ to 10 ^−5^ is used for this step to ensure that no spurious, suboptimal alignments are taken into account ^4^. If this homology search does not find any homologs outside of the analyzed species, the query gene has successfully been confirmed to be a *novel* gene. This definition states only that there are no homologous sequences outside of a certain phylogenetic group. Calling a gene novel does not imply any knowledge about the emergence mechanism of the gene.

To additionally determine *de novo* gene origin, the homologous non-coding outgroup DNA sequence has to be retrieved ^14, 23^. The outgroup homologous sequence can be recovered using synteny information about the position of orthologous neighbor genes. Another possibility is searching the target gene sequence in outgroup genomes using alignment search tools such as BLAST ^4, 23^. A number of different types of *de novo* genes can be discriminated depending on the type of sequence that the genes likely emerged from ^23^.

***Problems in de novo gene identification and annotation.*** In the past ^24^ and also more recently ^25, 26^, studies have raised questions regarding the reliability of homology-based searches of novel genes. Specifically, short and fast-evolving genes were proposed to lose detectable sequence similarity faster than other genes. As a result, shorter genes would be expected to be over-represented among young genes, thereby biasing the results of studies of genes of different ages ^24– 26^. Doubts have been raised as to which fraction of genes would actually be affected by this effect ^27^. Also, this should not be a problem for *de novo* genes defined by the methods summarized here. The possibility that the examined gene is actually a fast-evolving old gene is excluded, since for a confirmed *de novo* gene the homologous non-genic outgroup sequence has to be determined. Additionally, doubts have been raised regarding the accuracy of the initial claims of the unreliability of homology detection ^28^.

Another challenge is the previously mentioned identification of a non-coding sequence in an outgroup which is clearly homologous to the suspected *de novo* gene. In non-coding DNA, homology signals disappear very quickly, since non-coding sequences accumulate mutations faster than coding sequences. Because of this, it is often impossible to determine the homologous non-coding sequence in an outgroup. This problem increases with gene age. As a result, it is often not possible to determine the mechanism of origin, especially for older genes.

Additionally, there are methodological difficulties in the annotation of *de novo* and also all other types of novel genes ^4^. These problems could lead to a systematic underestimation of the number of *de novo*/novel genes. The problems are caused by genome annotation also being based on sequence homology ^29^. As *de novo*/novel proteins per definition do not possess any homologs, they cannot be annotated based on that criterion and their number is likely to be underestimated. Other common criteria such as minimum expression strength and the presence of multiple exons could also contribute to the problem, as these criteria do not represent intrinsic requirements for gene existence and are biased against *de novo*/novel genes ^18^. Nevertheless, the criteria might be necessary to prevent an over-annotation of spurious transcripts as genes, but they also make it impossible to identify all *de novo* genes. Recent studies on *de novo* protein-coding genes also employed such thresholds on exon number and expression strength to produce a more robust data set ^15, 17, 18^.

### *De novo* gene emergence

Conceptually, *de novo* genes can evolve via two different mechanisms. The first mechanism is transcription-first, where an intergenic sequence gains transcription before evolving an ORF ^20, 30^. Recently, this has been shown to happen frequently when long non-coding RNAs (lncRNAs) become protein coding ^17, 31, 32^. Consequently, lncRNAs could represent an intermediate step in the evolution of a protein-coding gene ^33^. The second model is ORF-first, in which an intergenic ORF gains transcription ^20, 30^. Such a transcribed *de novo* ORF has been proposed to represent an intermediate step in gene emergence, a protogene ( Figure 1). High turnover of intergenic transcription ^34^ likely plays a role in *de novo* gene emergence by exposing novel transcripts to selection. Transposable elements can also play a role in *de novo* gene emergence ^35^. Additionally to whole proteins, terminal domains can also emerge *de novo* ^33, 36^. One model regarding the emergence of novel domains is the “grow slow and molt”, in which reading frames get extended gradually and eventually gain a structure and function ^37, 38^.

An additional process that could play a role during *de novo* protein-coding gene emergence is a (partial) revival of pseudogenized gene fragments. This possibility has already been proposed by Ohno ^1^. Regarding *de novo* protein-coding gene emergence, it seems possible that fragments of a pseudogenized gene that has been somewhat eroded by drift could become part of a *de novo* ORF later on. These fragments could provide a starting point for *de novo* protein emergence by providing remnants of structural elements. For all of these models, there are several consistent findings, but none of the models is, as yet, supported by a comprehensive set of data from diverse sources and corresponding experimental data.

***De novo gene death.*** Orphan genes seem to generally have a high loss probability ^14, 39^ that seems to be negatively correlated with gene age ^40, 41^. The cause of this correlation is not yet well understood. It seems possible that young orphan genes have not yet gained a function or do not perform transient functions. It is also not clear yet how much of these findings can be transferred to *de novo* genes, as the studies on this topic examined all novel genes of different emergence mechanisms jointly.

### *De novo* gene functions

A number of studies have examined the functions of orphan genes, some of which may represent *de novo*-emerged genes. Findings on orphan gene functions include involvement of orphan genes in the stress response ^21, 42^, rapid adaptation to changing environments as well as species-specific adaptations ^43, 44^, and limb regeneration ^45^. Additionally, novel genes were found to quickly gain interaction partners and become essential ^39, 46^.

Fewer studies, however, have examined the functions of systematically verified *de novo*-emerged genes. Generally, a high number of *de novo* genes was found to be expressed specifically in the testes, at least in *Drosophila* species ^5, 6^ and primates ^18^, as well as in plant pollen ^16, 47^. In the mouse, a *de novo*-emerged RNA gene was found to raise reproductive fitness ^22^. Another study found *de novo* genes to play a role in the *Arabidopsis* stress response ^12^. More specifically, one *de novo* ORF was found to play a role in male reproduction in *Drosophila* ^48^. Reinhardt *et al.* ^48^ also presented findings suggesting a role of *de novo* genes in developmental stages of *Drosophila*. However, these findings have to be interpreted carefully, as the RNAi method used has been shown to produce unreliable results ^49, 50^. A few other examples of functional *de novo* genes have been found ^30^, while others were not able to determine specific functions of identified *de novo*-emerged genes ^15^. The available data suggest that *de novo*-evolved genes can play a role in many different processes from reproduction to the stress response.

Recently, one study analyzed the function of two putative *de novo* protein-coding genes in *Drosophila melanogaster* ^51^. The two analyzed genes were found to be essential for male reproduction and to have testis-biased expression. Both genes are located inside introns of other, older genes with homologs in outgroups. However, the *de novo* origin of the analyzed genes could not be confirmed with certainty owing to the outgroup homologous sequences not being identifiable (see above for a general description of this problem).

### Protein structure of *de novo* proteins

Little is known about the protein structures of *de novo* proteins. Some studies have found a high amount of intrinsic protein disorder ^52^ in very young genes ^15, 51, 53^, while others have not ^21^. *A priori*, it seems unlikely that *de novo*-emerging proteins have a well-defined protein structure. Intuitively, it seems more likely for random sequences to be intrinsically disordered instead (see Figure 1). Nevertheless, disordered regions can also be highly functional ^52, 54^ and could as such also represent an evolved state.

Also, contrary to intuition, at least semi-random (restricted alphabet) proteins appear to sometimes have a defined secondary structure ^55, 56^. Additionally, the existing protein structure families appear to have multiple origins ^57^. This finding suggests that the emergence of new protein structures is at least possible. Avoidance of misfolding and aggregation, on the other hand, have been proposed to be driving forces of protein evolution ^58, 59^. This observation and the existence of *de novo* protein-coding genes suggest that *de novo* proteins have the potential to exhibit a defined structure.

### Open questions regarding *de novo* genes

Despite many advances in recent years, many open questions remain regarding *de novo* protein-coding genes. One understudied field is the functional characterization of protein-coding *de novo*-emerged genes. One non-coding RNA gene has been found to have a role in reproduction in the mouse ^22^, and additionally one likely protein-coding gene has been found to be essential for reproduction in *Drosophila* ^48^. However, beyond that, there is a substantial lack of data. Consequently, it remains unclear how *de novo* protein-coding genes gain their function and if there are some roles that they are more or less likely to carry out.

As described above, the structural characterization of *de novo* protein-coding genes is still an open question. Previously, ambiguous signals have been found regarding the role of intrinsic disorder in *de novo*-emerging protein-coding genes ^15, 21^. It would be important to experimentally verify the structure — or lack thereof — of *de novo* protein-coding genes. Here it is of major interest to determine the proportion of intergenic ORFs with folding potential and also what the implications are for the retention of such ORFs. This would allow further conclusions about *de novo* gene emergence: if most intergenic, random ORFs are foldable, function would seem to be the bottleneck of *de novo* protein-coding gene retention. On the other hand, if most confirmed *de novo* genes are folding, but most intergenic ORFs do not possess folding potential, folding potential would be a bottleneck of *de novo* protein-coding gene emergence and retention.

Another unsolved problem is how to find specific annotation thresholds for orphans/ *de novo* genes ^4^. As described above, a number of their properties make *de novo* genes difficult to annotate and to be distinguished from transcriptional noise. One solution would be to generate high-quality proteome data using e.g. mass spectrometry. However, this process is still highly expensive and might also not be able to generate a complete picture, since low-frequency peptides are hard to detect ^60^. Another method is ribosome profiling, which uses ribosome occupancy of sequences as a measure of translation. This method has been successfully used to show that some transcripts that were previously classified as non-coding could in fact be translated ^61^.

Additionally, patterns of selection, e.g. measured in the ratio of non-synonymous to synonymous mutations, can be used to infer the coding status of sequences. Genes with a higher fraction of synonymous mutations compared to non-synonymous mutations can be expected to be protein coding and under purifying selection ^17, 20^. However, these measures require a number of orthologs to be present, which makes them of limited use for novel genes. Another possibility is the use of population data for the same purpose, which circumvents the problem of the unavailability of orthologs for novel genes.

As it stands, studies mostly have to rely on arbitrary cutoffs ^15, 17^ and thus might miss a number of genes. It would be of major interest to be able to differentiate *de novo* genes and protogenes from transcriptional noise. Recent research has already shown that small ORFs (smORFs) can play a functional role ^62, 63^, and consequently it seems quite likely that also very short novel ORFs could be functional. This question also touches upon the problem of differentiating lncRNAs from protein-coding genes, which is often performed via an ORF length cutoff ^17, 32^.

Going forward, it is of major interest to fully characterize a large number of *de novo* genes in terms of evolutionary, functional, and structural history to be able to draw some general conclusion about their evolution. Specifically, it is of major interest to determine whether a functional role is an exception for protogenes or if most expressed ORFs have a functional impact which mostly does not affect the fitness of the organism at a significant level. If most expressed ORFs have only a negligible fitness effect, they would mostly evolve via drift. Two closely related questions are how and when *de novo* proteins gain their function: are *de novo* genes usually functional from the time point of their emergence, or do they gain a cellular task only after a period of drift?

## Conclusions

In recent years, an increasing number of studies confirmed a major role of *de novo* gene emergence in the evolution of new protein-coding genes. The functional description of *de novo*-emerged genes is still lacking, but more general findings for orphan genes suggest that novel genes have a broad functional potential. However, the more detailed functional as well as structural characterization of *de novo*-emerged protein-coding genes remains one of the big open questions. An interesting recent finding was the confirmation of lncRNAs as an intermediate step in *de novo* protein-coding gene evolution. This finding offers a solution to two of the big questions in *de novo* gene evolution — how and why do intergenic sequences gain transcription? However, these findings also touch upon a difficult problem in studying *de novo* genes: how can protein-coding genes be distinguished from non-coding ones? This problem is exacerbated by recent findings that show that very short ORFs can also be functional ^63^. Tackling all of these problems and integrating them into detailed studies of the emergence, structure, and function of *de novo* protein-coding genes will provide new, interesting insights and allow for a deeper understanding of the inner workings of the evolution of *de novo* protein-coding genes.
